# Clinical Manifestations and Case Management of Ebola Haemorrhagic Fever Caused by a Newly Identified Virus Strain, Bundibugyo, Uganda, 2007–2008

**DOI:** 10.1371/journal.pone.0052986

**Published:** 2012-12-28

**Authors:** Paul Roddy, Natasha Howard, Maria D. Van Kerkhove, Julius Lutwama, Joseph Wamala, Zabulon Yoti, Robert Colebunders, Pedro Pablo Palma, Esther Sterk, Benjamin Jeffs, Michel Van Herp, Matthias Borchert

**Affiliations:** 1 Medical Departments of Médecins Sans Frontières, Barcelona, Spain; 2 Medical Departments of Médecins Sans Frontières, Geneva, Switzerland; 3 Medical Departments of Médecins Sans Frontières, Brussels, Belgium; 4 Department of Global Health and Development, London School of Hygiene & Tropical Medicine, London, United Kingdom; 5 Medical Research Council Centre for Outbreak Analysis and Modelling, Department of Infectious Disease Epidemiology, Imperial College London, London, United Kingdom; 6 Department of Arbovirology, Emerging, and Re-Emerging Viral Infections, Uganda Virus Research Institute, Entebbe, Uganda; 7 National Disease Control, Uganda Ministry of Health, Kampala, Uganda; 8 Disease Surveillance and Response Cluster, World Health Organization Regional Office for Africa, Kampala, Uganda; 9 Department of Clinical Sciences, Institute of Tropical Medicine Antwerp and Department of Epidemiology and Social Medicine, University of Antwerp, Antwerp, Belgium; 10 Department of Infectious Disease Epidemiology, London School of Hygiene & Tropical Medicine, London, United Kingdom; 11 Institute of Tropical Medicine and International Health, Charité-Universitätsmedizin Berlin, Berlin, Germany; University of Liverpool, United Kingdom

## Abstract

A confirmed Ebola haemorrhagic fever (EHF) outbreak in Bundibugyo, Uganda, November 2007–February 2008, was caused by a putative new species (*Bundibugyo ebolavirus*). It included 93 putative cases, 56 laboratory-confirmed cases, and 37 deaths (CFR = 25%). Study objectives are to describe clinical manifestations and case management for 26 hospitalised laboratory-confirmed EHF patients. Clinical findings are congruous with previously reported EHF infections. The most frequently experienced symptoms were non-bloody diarrhoea (81%), severe headache (81%), and asthenia (77%). Seven patients reported or were observed with haemorrhagic symptoms, six of whom died. Ebola care remains difficult due to the resource-poor setting of outbreaks and the infection-control procedures required. However, quality data collection is essential to evaluate case definitions and therapeutic interventions, and needs improvement in future epidemics. Organizations usually involved in EHF case management have a particular responsibility in this respect.

## Introduction

*Filoviridae* family members are characterised by filamentous enveloped particles with a negative-sense single-stranded RNA genome. They are divided into two genera, *Ebolavirus* and *Marburgvirus*, respectively causing Ebola and Marburg haemorrhagic fever (EHF, MHF) in human and non-human primates [1]. Filovirus haemorrhagic fever (FHF) outbreaks are characterised by secondary transmission and high case fatality [2], [3], although species-specific case fatality ratios (CFR) vary considerably: *Zaire ebolavirus* (ZEBOV; 80–90%) [2], [4], [5], *Sudan ebolavirus* (SEBOV; 40–65%) [2], *Bundibugyo ebolavirus* (BEBOV; 25%) [6]–[8], *Côte d'Ivoire ebolavirus* (CIEBOV; 0%; based on a single patient) [9]–[11], *Reston ebolavirus* (REBOV; 0%; possibly non-pathogenic for humans) [12]–[16], and *Lake Victoria marburgvirus* (MARV; 20–88%) [17]–[20]. To date, 35 FHF outbreaks are known to have occurred in humans (24 EHF and 11 MHF), all in or originating from sub-Saharan Africa [2], [21]–[27].

### Suspect and laboratory-confirmed patient categorisation

In sub-Saharan Africa, when a medical professional suspects a filovirus infection, the patient's blood sample is typically sent abroad to a biosafety level-4 (BSL-4) laboratory for diagnostic confirmation. If positive, an outbreak is declared and an international response initiated, consisting of case identification and contact tracing, with isolation and treatment of suspect and laboratory-confirmed patients in a filovirus ward [28]–[33].

Due to delays between outbreak onset, recognition, and response, some individuals potentially infected with filovirus convalesce, die and are buried, or are lost to follow-up before having their blood sampled for disease confirmation. Likewise, if clinical disease and outcome occur prior to a filovirus ward's existence or functionality, not all patients are hospitalised and treated on a filovirus ward. Outbreaks therefore habitually conclude with putative, suspected, and laboratory-confirmed patient categorisations, with only some patients receiving supportive treatment on a filovirus ward.

Once case identification and contact tracing activities commence, individuals matching epidemiological and clinical case definitions are accompanied to a filovirus ward for clinical assessment and, when appropriate, categorised as a suspected FHF patient while a blood sample is drawn and sent for laboratory confirmation. Diagnostic results are typically available within four hours from an on-site laboratory, 48 hours from a laboratory elsewhere in the country, or a week for samples sent abroad [33], [34]. Patients with negative test results are discharged and assessed for an alternative illness or remain on the ward and are re-tested if FHF clinical suspicion remains. Laboratory-confirmed patients remain hospitalised on the filovirus ward until virus clearance and recovery or death.

### Standard case management

In the absence of specific antifiloviral therapy, filovirus ward clinicians provide suspect and laboratory-confirmed patients with the supportive care regime administered during the 1995 EHF outbreak in Kikwit, Democratic Republic of the Congo and subsequent outbreaks, consisting of oral medication, oral fluid rehydration, nutritional supplementation, and psychosocial support [26], [29]–[31]. Oral medication includes those that alleviate FHF-related symptoms such as nausea and vomiting (e.g. metoclopramide and promethazine), dyspepsia (e.g. aluminium hydroxide, cimetidine, ranitidine, and omeprazole), anxiety, agitation, or confusion (e.g. diazepam, chlorpromazine), and pain (e.g. paracetamol, tramadol, and morphine), when indicated. In addition to supportive care, oral artemether/lumefantrine for uncomplicated malaria and an oral antibiotic (e.g. amoxicillin, cotrimoxazole, cefixime, or ciprofloxacin) are uniformly administered due to the customary absence of an on-site laboratory capable of safely processing biological samples for alternative diagnoses. Recently expanded, supportive care may also include prevention and treatment of dehydration via intravenous (IV) fluids, nasogastric delivery of nutritional and vitamin supplementation, and IV administration of medication for optimum drug delivery when clinically indicated [26], [29].

### Improving knowledge of human clinical manifestations and case management

Limited quality FHF clinical data from human outbreaks have been collected, analysed, and published, partly due to safety concerns about transferring paper-based clinical records outside the filovirus ward [26]. Records have been destroyed as potential fomites, not recorded, or haphazardly logged [26]. As a result, most detailed descriptions of clinical manifestation have been from laboratory-based studies of non-human primates [35]–[39] and a limited number of human patients (e.g. ZEBOV [28], [40]–[52], SEBOV [53]–[56], CIEBOV [9], and MARV [19], [33], [57]–[67]).

Substantial uncertainties remain regarding human FHF incubation periods and symptom frequency, onset, and duration. Retrospectively collected FHF clinical data are of questionable validity and reliability due to reporting and recall biases [41], [48], [49], [53], [54]. Although some outbreak analyses have yielded symptom frequency and duration [9], [19], [40], [49], [53], [55], numerous others yielded only frequency data [24], [28], [33], [41], [45]–[48], [54]. Point and period prevalence of symptoms (e.g. at admission to the filovirus ward, during hospital stay) fail to document the clinical course of disease. Understanding human FHF symptomatology is crucial for advancing outbreak control measures and administering supportive treatment based on symptom presentation and disease severity [33].

Although anecdotal evidence suggests supportive treatment increases FHF survival, its effectiveness has not been assessed in an outbreak setting [21], [29], [46]. More data on human clinical manifestations and treatment effectiveness are needed to improve response to these poorly understood diseases.

### The 2007–2008 Bundibugyo outbreak

On 29 November 2007, the Uganda Ministry of Health (MoH) and the World Health Organization (WHO) confirmed an outbreak of EHF in Bundibugyo District, western Uganda, and responded in collaboration with Médecins Sans Frontières (MSF), the Uganda Virus Research Institute (UVRI), the United States Centers for Disease Control and Prevention (CDC), and others [68]. On 20 February 2008, the outbreak concluded with 93 putative and 56 laboratory-confirmed cases (30 of whom were hospitalised) and 37 deaths, yielding a 25% CFR [69], [70]. Most cases originated from Bundibugyo and Kikyo, towns of approximately 16,000 and 5,700 inhabitants respectively. Bundibugyo is situated at the base of the Rwenzori Mountains, and Kikyo 25 kilometres within them. An Ebola ward was set up and maintained at each location throughout the outbreak response [70]. Genetic sequencing of viral RNA, conducted at CDC Atlanta, confirmed that the virus causing the Bundibugyo outbreak differed from any known ebolavirus (EBOV) species and was, although most closely related to CIEBOV, therefore proposed as a new EBOV species provisionally named *Bundibugyo ebolavirus* (BEBOV) [71].

### Study rationale and objectives

This outbreak is the first known observation of human disease caused by this putatively novel EBOV species. Thus, documenting clinical manifestations of BEBOV infection furthers knowledge of human FHF symptomatology, while describing the implemented FHF case management strategy identifies its merits and shortcomings, a baseline imperative for improving and assessing the effectiveness of supportive case management. Pending antifiloviral therapy development, this may be the only way for future patients to receive better care. The objectives of this secondary analysis of patient data are to (i) describe patient demographics and contact histories; (ii) document symptoms from onset to clinical outcome; (iii) describe case management on the Ebola ward; and (iv) recommend strategies for improving data collection in future FHF outbreaks.

## Methods

### Ethics statement

The Uganda National Health Research Organization and the Ethics Review Boards of Médecins Sans Frontières and London School of Hygiene & Tropical Medicine provided ethics approval for *a posteriori* analyses of the outbreak's anonymous and routinely collected clinical and epidemiological data. As no additional data were collected for research purposes and all data were anonymous before analysis, the ethical review boards waived the need for patients' consent.

### Study population and data collection

Study subjects were patients hospitalised on Bundibugyo or Kikyo Ebola wards with subsequent EHF laboratory confirmation. The case definitions to identify suspected EHF cases in Bundibugyo District were: (i) an epidemiological link to an individual potentially infected with EBOV and at least three general symptoms (i.e. asthenia, anorexia, myalgia/arthralgia, diarrhoea, abdominal pain, nausea, vomiting, headache, dysphagia, dyspnoea, conjunctivitis, jaundice, hiccups); or (ii) fever plus at least three of the general symptoms listed above; or (iii) fever plus unexplained haemorrhage. Individuals fulfilling one or more definitions were accompanied to an Ebola ward and clinically assessed. Suspected cases further corroborated by clinical assessment had a venipuncture-acquired blood sample drawn and sent to the UVRI/CDC laboratory in Entebbe, Uganda for biological confirmation by polymerase chain reaction (PCR), antigen detection by enzyme-linked immunosorbent assay (ELISA), or IgM-capture ELISA [69], [71]. Laboratory results were available three to five days after sample extraction.

**Figure 1:**
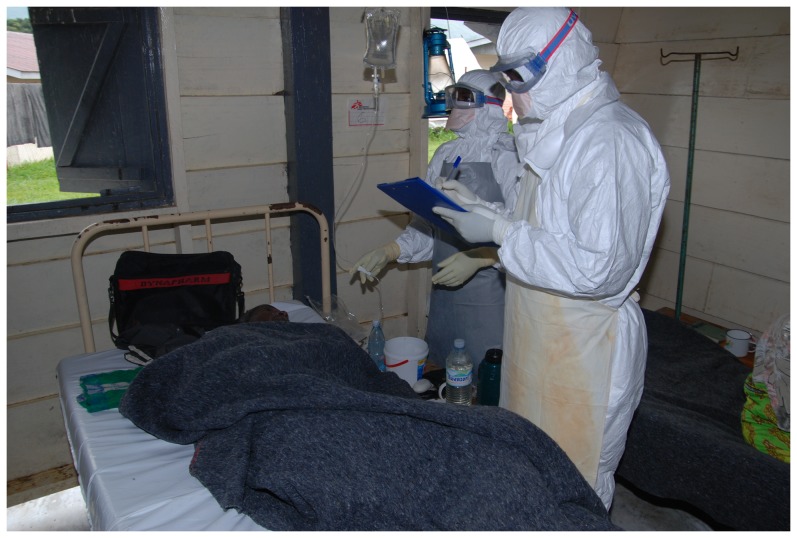
Filovirus ward clinicians administering supportive treatment while concurrently recording clinical data during the Bundibugyo Uganda 2007–08 Ebola haemorrhagic fever outbreak. Photo by Claude Mahoudeau.

The makeshift Bundibugyo and Kikyo Ebola wards became fully functional following implementation of WHO and MSF infection control and treatment protocols [3], [31]. Study subjects were hospitalised on (i) a makeshift ward for the entirety of their stay; (ii) a makeshift ward that became fully functional during their stay; or (iii) a fully functional ward for the entirety of their stay. Differences between fully functional and makeshift wards were additional medical supplies, case management as described in the introduction, and standardised data collection [48]. Data were recorded by MoH staff on makeshift wards and MoH, WHO, and MSF staff on fully functional wards (Figure 1).

### Study variables and data analysis

Data were collected on patient demographics, contact history, symptoms (self-reported from disease onset until presentation at the Ebola ward or observed by healthcare workers from admission until clinical outcome), treatment, patient monitoring, and clinical outcome.

Demographic variables were age, gender, residence, occupation, and Ebola ward. Contact history with an individual potentially infected with EBOV was categorized as none, indirect (i.e. via fomite), direct, and direct during funeral practices [33]. Symptoms were categorised as general or haemorrhagic. Supportive treatment was categorised as EHF-related symptom alleviation, antibiotics, antimalarials, and dehydration management. Nutritional and psychosocial support data were not collected. Patient monitoring data were axillary body temperature (i.e. fever defined as axillary body temperature ≥38.0° Celsius), heart rate (beats per minute), respiratory rate (breaths per minute), and blood pressure (mmHg). Clinical outcome was defined as death or survival on discharge from the Ebola ward.

Data were analysed using Microsoft Excel and Stata® 11.0 (StataCorp Texas) to describe symptom frequencies, duration, and pattern and to determine associations between demographics, symptoms, or treatment and clinical outcome using exact logistic regression for small samples. Simple binomial logistic regression was used to determine probability of death by number of symptoms. Fisher's exact p-values of <0.05 were considered statistically significant.

## Results

Our study sample included 26 of the 30 hospitalised laboratory-confirmed EHF patients. Four patients were excluded for lacking symptom and/or clinical outcome data. Self-reported symptoms were recorded for 15 patients, clinically observed symptoms for 21 patients, and treatment details for 19 patients.

### Demographics and contact history

Table 1 presents self-reported demographics, days before seeking treatment at an Ebola ward, and contact histories of the 26 patients, stratified by clinical outcome. Median age was 37 years (range 20–66) and 73% were male. No female patients were known to be pregnant, though one had miscarried the day prior to her Ebola ward admission and another was breastfeeding. Most (73%) were isolated in the Bundibugyo Ebola ward. Half of patients (6/12; 50%) with occupation recorded were health workers, and five of these (83%) were male. All 14 patients (100%) with a recorded contact history reported direct contact and/or contact during funeral practices, whereas 12 patients (38%) reported no known contact. Demographic and contact history variables were not associated with clinical outcome (Fisher's exact p-value range: 0.25–0.69).

**Table 1:** Self-reported demographics, days before seeking treatment at an Ebola ward, and contact histories of 26 patients with laboratory-confirmed Ebola haemorrhagic fever, Bundibugyo District, Uganda (November 2007–February 2008).

Characteristics	Survivedn = 15 (row%^1^)	Diedn = 11 (row%^1^)	Totaln = 26 (col%^1^)	Odds ratio (95%CI)^2^ *
Median age (range)	35 (21–50)	39 (20–66)	37 (20–66)	1.03 (0.96–1.11)
Gender				
Female	4 (57)	3 (43)	7 (27)	baseline
Male	11 (58)	8 (42)	19 (73)	0.97 (0.12–8.56)
Occupation				
Health worker	3 (50)	3 (50)	6 (23)	baseline
Farmer	0 (0)	2 (100)	2 (8)	1.68 (0.11-inf)
Other	3 (75)	1 (25)	4 (15)	0.88 (0.51–1.31)
Unknown	9 (64)	5 (36)	14 (54)	0.99 (0.97–1.02)
Days before seeking treatment at Ebola ward^3^				
0–3 days symptomatic	9 (69)	4 (31)	13 (50)	baseline
4–8 days symptomatic	5 (56)	4 (44)	9 (35)	1.75 (0.22–14.6)
Unknown	1 (25)	3 (75)	4 (15)	1.29 (0.86–2.35)
Ebola ward				
Bundibugyo ward	11 (58)	8 (42)	19 (73)	baseline
Kikyo ward	4 (57)	3 (43)	7 (27)	1.03 (0.12–8.14)
Symptoms data records				
Only self-reported	3 (60)	2 (40)	5 (19)	baseline
Only clinically-observed	6 (60)	4 (40)	10 (39)	1.0 (0.07–17.3)
Both recorded	6 (55)	5 (45)	11 (42)	1.11 (0.31–4.53)
Contact history				
No known contact	7 (58)	5 (42)	12 (46)	baseline
Known contact	8 (57)	6 (43)	14 (54)	1.16 (0.18–8.00)
Direct (non-funeral)	6 (55)	5 (45)	11 (79)	..
Direct (funeral practices)	2 (67)	1 (33)	3 (21)	..

NB:

* Results significant at p<0.05.

1 Except age, where brackets include the range.

2 OR calculates odds ratio for fatal outcome and 95% confidence intervals, comparing exposed to reference (baseline or OR = 1) patients, using exact methods and Fisher's exact p-values for small sample sizes (confounders have not been adjusted for due to small cell sizes).

3 Patient's villages were all located within a one-hour walk of Bundibugyo or Kikyo towns.

### Course of disease and clinical manifestations

Eleven of the 26 patients died (CFR = 42%). The median duration of disease was 9 days (range 3 to 20) from self-reported symptom onset to death for 11 patients and 9.9 days (range 2 to 21) from self-reported onset to last recorded symptom prior to discharge for 15 surviving patients. Patients presented to an Ebola ward after a mean self-reported delay of 3.5 days (range 0 to 8) following symptom onset. Available data do not indicate that delayed hospitalisation increased probability of death (e.g. differences were not significant for patients who died after being admitted four to eight days (4/9; 44%) versus zero to three days (4/13; 31%) after symptoms reportedly commenced [Fisher's exact p-value 0.66; Table 1]).

Table 2 presents all recorded patient symptoms. The left side shows frequency, usual day of onset, and mean duration in days of self-reported symptoms among 15 patients. The most frequently reported general symptoms were fever, nausea/vomiting and non-bloody diarrhoea (11/15; 73% each), abdominal pain (9/15; 60%), and conjunctivitis (5/15; 33%). Each self-reported symptom was experienced for a median of three to four days prior to hospitalisation. Although no individual self-reported symptom was associated with clinical outcome (Fisher's exact p-value range 0.23–1.00), each additional self-reported symptom significantly doubled the odds of death (OR 2.14; 95%CI: 1.02–8.18).

**Table 2:** Self-reported symptoms (15 patients), clinically observed symptoms (21 patients), and combined symptoms (26 patients) among hospitalised laboratory-confirmed EHF patients with known clinical outcome for whom data were recorded, Bundibugyo District, Uganda (November 2007–February 2008).

	Self-reported symptoms (15 patients) from onset to admission			Clinically observed symptoms (21 patients) from admission to clinical outcome				Self-reported or clinically observed symptoms (26 patients) from onset to clinical outcome		
Symptoms	Survivedn = 9 (%)	Diedn = 6 (%)	Usual day of onset (mean duration in days)	Survivedn = 12 (%)	Diedn = 9 (%)	Pattern(cont/int)*	Mean duration in days(range in days)	Survivedn = 15 (%)	Diedn = 11 (%)	Totaln = 26 (%)
*General (Any)*	*9 (100)*	*6 (00)*		*12 (100)*	*9 (100)*	*–*	*–*	*15 (100)*	*11 (100)*	**26 (100)**
Non-bloody diarrhoea	5 (56)	6 (100)	1 (4.1)	7 (58)	7 (78)	4/12	4 (1–9)	10 (67)	11 (100)	21 (81)
Severe headache	2 (22)	2 (33)	1–6 (3.0)	12 (100)	8 (89)	17/1	7 (1–11)	12 (80)	8 (81)	21 (81)
Asthenia	3 (33)	1 (17)	1 (4.7)	10 (83)	8 (89)	18/0	7 (1–13)	11 (73)	9 (82)	20 (77)
Nausea/Vomiting	5 (56)	6 (100)	1 (3.5)	5 (42)	8 (89)	9/2	3 (1–11)	7 (47)	10 (91)	17 (65)
Myalgia	3 (33)	0 (0)	1 (3.0)	8 (67)	8 (89)	14/2	8 (2–11)	9 (60)	8 (73)	17 (65)
Abdominal pain	4 (44)	5 (83)	1 (4.1)	5 (42)	7 (78)	10/1	3.5 (1–9)	7 (47)	9 (82)	16 (62)
Dysphagia	0 (0)	2 (33)	1–2 (2.0)	7 (58)	8 (89)	12/2	8 (2–11)	7 (47)	8 (73)	15 (58)
Appetite loss	0 (0)	1 (17)	1 (2.0)	7 (58)	8 (89)	14/0	5 (1–10)	7 (47)	8 (73)	15 (58)
Conjunctivitis	2 (22)	3 (50)	1 (3.8)	5 (42)	4 (44)	9/0	7 (3–9)	7 (47)	6 (55)	13 (50)
Fever	6 (67)	5 (83)	1 (3.6)	0 (0)	1 (11)	0/1	1	6 (40)	5 (45)	11 (42)
Non-bloody rash	1 (11)	2 (33)	1 (3.7)	0 (0)	0 (0)	0/0	0 (0)	1 (7)	2 (18)	3 (12)
Chest pain	0 (0)	0 (0)	–	2 (17)	5 (56)	5/1	4 (1–9)	2 (13)	5 (45)	7 (27)
Dyspnea	0 (0)	0 (0)	–	1 (8)	4 (44)	3/0	3 (1–5)	1 (7)	4 (36)	5 (19)
Lumbar pain	0 (0)	0 (0)	–	1 (8)	3 (33)	3/0	2.5 (1–9)	1 (7)	3 (33)	4 (17)
Cough	0 (0)	0 (0)	–	3 (25)	1 (11)	4/0	5 (3–7)	3 (20)	1 (9)	4 (15)
Disorientation	NA	NA	–	0 (0)	4 (44)	1/0	3 (3)	0 (0)	4 (36)	4 (15)
Anuria	0 (0)	0 (0)	–	0 (0)	2 (22)	2/0	1.5 (1–2)	0 (0)	2 (18)	2 (8)
Dehydration	NA	NA	–	0 (0)	2 (22)	1/0	2 (2)	0 (0)	2 (18)	2 (8)
Hiccoughs	0 (0)	0 (0)	–	0 (0)	1 (11)	1/0	1 (1)	0 (0)	1 (9)	1 (5)
Right upper-quad. pain	0 (0)	0 (0)	–	0 (0)	1 (11)	1/0	5 (5)	0 (0)	1 (9)	1 (5)
Haemorrhagic (Any)	1 (11)	2 (33)	–	1 (8)	4 (44)	–	–	1 (7)	6 (55)	7 (27)
Melaena	0 (0)	1 (17)	1 (3)	0 (0)	2 (22)	1/1	3 (2–4)	0 (0)	3 (27)	3 (12)
Epistaxis	1 (11)	1 (17)	2–7 (1)	1 (8)	0 (0)	0/1	1 (1)	1 (7)	1 (9)	2 (8)
Haematemesis	0 (0)	1 (17)	2 (1)	0 (0)	1 (11)	1/0	1 (1)	0 (0)	2 (18)	2 (8)
Injection bleeding	0 (0)	0 (0)	–	0 (0)	2 (22)	1/0	1 (1)	0 (0)	2 (18)	2 (8)
Postpartum bleeding	0 (0)	1 (17)	2 (1)	0 (0)	1 (11)	1/0	1 (1)	0 (0)	1 (9)	1 (4)
Haemoptysis	0 (0)	0 (0)	–	0 (0)	1 (11)	1/0	1 (1)	0 (0)	1 (9)	1 (4)
Bleeding gums	0 (0)	0 (0)	–	0 (0)	1 (11)	1/0	1 (1)	0 (0)	1 (9)	1 (4)
Haematuria	0 (0)	0 (0)	–	0 (0)	1 (11)	1/0	3 (3)	0 (0)	1 (9)	1 (4)
Haematoma	0 (0)	0 (0)	–	0 (0)	1 (11)	1/0	1 (1)	0 (0)	1 (9)	1 (4)

* Continuous/Intermittent.

The centre of Table 2 presents frequency, pattern (continuous versus intermittent), and mean duration in days of clinically observed symptoms among 21 patients, from presentation to the Ebola ward until clinical outcome. Frequent symptoms included severe headache (20/21; 95%), asthenia (18/21; 86%), myalgia (16/21; 76%), dysphagia and appetite loss (15/21; 71% each), and non-bloody diarrhoea (14/21; 67%). Each symptom lasted a mean duration of 3.5–8 days (range 1–13). Less frequent clinically observed general symptoms (conjunctivitis, chest pain, cough, and right upper-quadrant pain) had relatively protracted duration (mean 4–7 days). Of ten patients whose body temperature was recorded at least once while hospitalised, one patient (10%) developed fever for one day.

**Figure 2:**
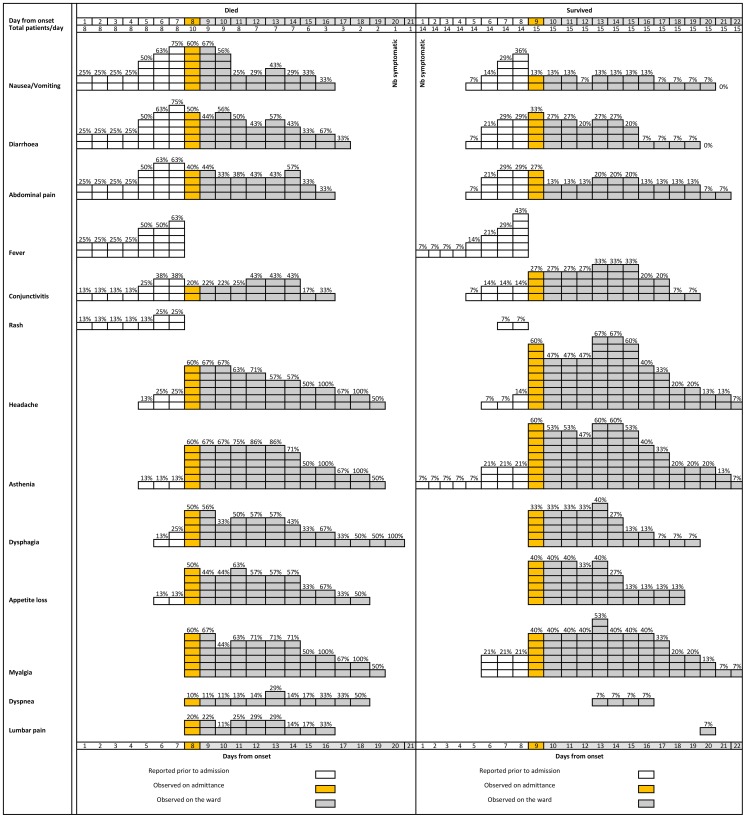
Frequency of non-haemorrhagic symptoms from self-reported day of symptom onset to clinical outcome, as absolute numbers and percentages, among symptomatic (9 deceased and 12 surviving) laboratory-confirmed Ebola haemorrhagic fever patients, Bundibugyo District, Uganda, November 2007–February 2008. Note changes in denominator between self-reported and clinically observed sections.

**Figure 3:**
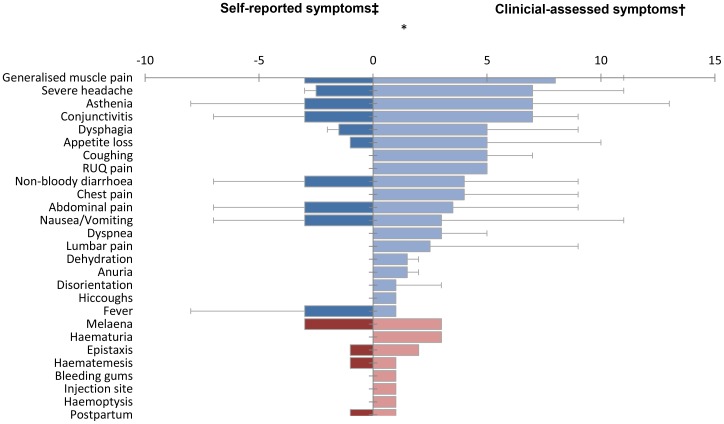
Median duration in days of symptoms from self-reported onset until clinical outcome among 26 symptomatic laboratory-confirmed Ebola haemorrhagic fever patients, Bundibugyo District, Uganda (November 2007–February 2008). Blue and red bars indicate general and haemorrhagic symptoms, respectively. *Day 0 = presentation to the Ebola ward. ^‡^Whiskers indicate maximum duration of the self-reported symptoms prior to presentation to the Ebola ward for patient observations >1. ^†^Whiskers indicate maximum duration of the clinician-assessed symptoms at presentation to and during hospitalisation on the Ebola ward for patient observations >1. ^#^Denominator contains female patients only (n = 9).

The right of Table 2 and Figure 2 present symptom frequency from self-reported onset to clinical outcome (i.e. self-reported and clinically observed) for the 26 study-patients. The most frequently experienced symptoms were non-bloody diarrhoea (81%), severe headache (81%), and asthenia (77%), while Figure 3 presents their median duration.

No self-reported or clinically observed symptom or combination of symptoms, other than any haemorrhage (Fisher's exact p-value 0.05), was associated with clinical outcome (Fisher's exact p-value range: 0.37–1.00). However, similarly to self-reported symptoms, for each additional clinically observed symptom, the odds of death increased by approximately 31% (OR 1.31; 95%CI: 1.04–1.82).

### Haemorrhagic symptoms

Seven patients experienced self-reported and/or clinically observed haemorrhagic symptoms, six of whom died (Table 2). Haemorrhagic patients had twelve times greater odds of dying than those not experiencing any haemorrhagic symptom (86% versus 33%; OR 12.0, exact 95%CI: 1.02–590; data not shown).

Three patients self-reported haemorrhagic symptoms prior to admission while five patients were clinically observed with haemorrhagic symptoms during hospitalisation. The one surviving patient self-reported and was clinically observed with epistaxis (Table 2). Of the two patients who self-reported haemorrhage and later died, one reported melaena, while the other reported haematemesis, epistaxis, and postpartum bleeding (Table 2). In neither patient were haemorrhagic symptoms clinically observed during hospitalisation. Of the three patients who self-reported a haemorrhagic symptom, two presented to an Ebola ward within 24 hours of self-reported bleeding onset, while one tolerated melaena for three days prior to presenting. Each of these three patients had a self-reported median of one day of fever prior to Ebola ward presentation.

**Figure 4:**
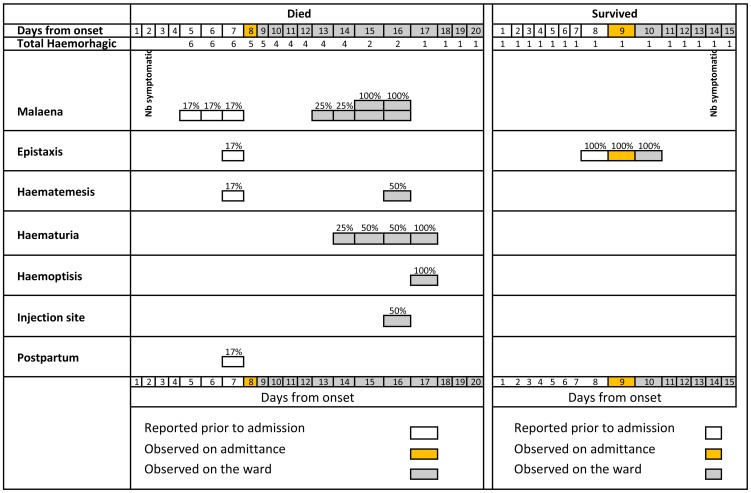
Frequency of haemorrhagic symptoms from self-reported day of symptom onset to clinical outcome, as absolute numbers and percentages, among symptomatic (9 deceased and 12 surviving) laboratory-confirmed Ebola haemorrhagic fever patients, Bundibugyo District, Uganda, November 2007–February 2008. Note changes in denominator between self-reported and clinically observed sections.

Clinically observed haemorrhagic symptoms (Table 2; Figure 4) included melaena and prolonged bleeding at an injection site (2/21; 10% each), and haematemesis, bleeding gums, haemoptysis, haematuria, haematoma, and postpartum vaginal bleeding (1/21; 5% each). Of the four patients who were only clinically observed with haemorrhagic symptoms, the first was observed with bleeding gums; the second with prolonged bleeding from an injection site; the third with melaena, haematemesis, and prolonged bleeding from an injection site; and the fourth with melaena, haemoptysis, haematuria, haematoma, and postpartum bleeding. Petechiae were not observed.

### Case management

Of the 19 laboratory-confirmed patients with treatment details recorded, 18 (95%) were administered paracetamol to alleviate pain and one patient (1%) received cimetidine for dyspepsia (Table 3). No other medication was administered to alleviate Ebola-related symptoms. Antibiotics were administered to seven patients (37%) for potential concomitant infections. Antimalarials were administered to 11 patients (58%), 73% of whom died during hospitalisation, yielding a borderline significant positive association between antimalarial administration and fatal outcome (OR 5.93, 95%CI: 0.93–50.5, Fisher's exact p-value = 0.05). However, two of these patients received quinine, indicating more severe infection. When these two were removed from analysis, and analysis was restricted to patients receiving presumptive artemether/lumefantrine, the difference in clinical outcome was no longer significant (p = 0.23). Oral rehydration solution (ORS) was administered to 16 patients (84%), while four patients (21%) received IV-fluids. Vitamin supplementation was not recorded. With the exception of antimalarial treatment, there was no significant difference in clinical outcome for any treatment component (Fisher's exact p-value range 0.33–1.00).

**Table 3:** Treatment recorded for 19 hospitalised laboratory-confirmed Ebola haemorrhagic fever patients, by clinical outcome.

Treatment	Survived (%)^1^	Died (%)^1^	OR (95%CI)^2^
*Any recorded treatment*	8 (47)	9 (53)	5.04 (0.54-inf)
*Pain relief*			
Paracetamol 3 grams/day	9 (50)	9 (50)	1.48 (0.25–9.82)
*Fluids*	9 (56)	7 (44)	0.78 (0.14–4.46)
ORS alone	9 (56)	7 (44)	..
ORS and Ringers lactate+GL 5% 3l	2 (100)	0 (0)	..
ORS and Ringers lactate+dextrose 5%2l	0 (0)	2 (100)	..
*Antimalarials*	3 (27)	8 (73)	5.93 (0.93–50.5)
Artemether/lumefantrine	3 (33)	6 (67)	..
Quinine	0 (0)	2 (100)	..
*Antibiotics*	3 (43)	4 (57)	1.74 (0.23–15.1)
Amoxicillin	1 (25)	3 (75)	..
Ciprofloxacin	2 (100)	0 (0)	..
Ceftriaxone	0 (0)	1 (100)	..
*Dyspepsia relief*			
Cimetidine	0 (0)	1 (100)	–

Bundibugyo District, Uganda, November 2007–February 2008.

NB:

1 Row percentages.

2 OR calculates the odds ratio for fatal outcome and 95% confidence intervals, comparing patients who received treatment to those who did not, using exact methods and Fisher's exact p-values for small sample sizes (confounders have not been adjusted for due to small cell sizes).

Of the ten patients whose axillary body temperature was recorded at least once during hospitalisation, five (50%) had their temperature recorded at least once daily for 80% of their stay, while seven (70%) had their temperature recorded at least once daily for 50% of their stay (data not shown). Heart rate, respiratory rate, and blood pressure were not recorded for any patients.

## Discussion

This study documents clinical manifestations of human BEBOV infection among hospitalised patients and describes case management strategy. Documenting clinical manifestations from a putatively novel EBOV species furthers knowledge of human filovirus infection, while describing case management identifies areas for improvement and accentuates the need to assess effectiveness of supportive treatment in future outbreaks.

### Case fatality ratio

To date, the 25% crude CFR of the 2007–2008 Bundibugyo outbreak is the lowest of recorded major human EHF outbreaks [8]. However, among hospitalised laboratory-confirmed patients the CFR increases to 42%, similar to that found by MacNeil and colleagues for laboratory-confirmed acute-phase samples [69] and observed consistently in SEBOV [53]–[55], [72] and occasionally in ZEBOV [41], [73] outbreaks. The low crude CFR could be biased by false positives among putative cases [8] or more accurate due to inclusion of less severe cases who did not attend hospital. Attributing differences in CFRs to specific filovirus species merits caution, as disease recognition often requires a functioning surveillance system and case-fatality is influenced by numerous factors beyond viral species, including route and dose of infection, genetic susceptibility, and underlying prevalence of immunodeficiency and co-morbid conditions [16], [49], [54].

### Patient demographics and contact histories

Similar to some previous filovirus outbreaks, this study population only comprised adults (20–66 years) [49], [74], [75]. All study patients reporting contact reported direct contact (11/14) or direct contact with a potentially infected corpse during funeral practices (3/14). Direct and funeral contacts were frequent opportunities for disease transmission in previous filovirus outbreaks [33], [49], [75]–[78]. However, 46% (12/26) of our study population reported no known contact history. This may be due to non-rigorous patient interviews during high-workload periods on the Ebola ward. Considerable amounts of routine data were missing (e.g. 54%, or 14/26 of study subjects did not have their occupation recorded), indicating shortcomings in data collection. Alternatively, primary or unnoticed secondary transmission could have occurred. Available data preclude decisive conclusions.

Regrettably, incubation periods were not measured for this study population. Albeit challenging to establish as an individual may have had prolonged contact with a source case, it is possible to obtain quality contact history and incubation-period data [9], [28], [40], [41], [46]–[49], [53], [55], [67], [69], [79]. Contact history data facilitate outbreak control efforts and further understanding of transmission patterns [33], while incubation time-period contributes to the understanding of disease course in humans [35], [80]. Without complete and accurate data, interpretation of demographic distribution and contact history is difficult. Authors are, for example, precluded from explaining why 73% of the study population were male.

### Clinical manifestations

Ebola-ward clinicians working on the fully functional ward employed a standardised prospective case reporting form for the Bundibugyo outbreak and prioritised data transfer outside the ward [19], [81], [82]. Subsequent analyses (Tables 2, 3; Figures 2 and 3) document the first recognized observation of human disease caused by this putatively novel EBOV species and further knowledge of FHF clinical manifestations and disease course. Symptoms previously observed and reported from human ZEBOV [28], [40]–[52], SEBOV [53]–[55], CIEBOV [9], and MARV [19], [33], [57]–[67] infections are congruous with these clinical data. A more detailed comparison is not feasible due to substantial variations in FHF reporting methodology.

As filovirus ward clinicians often use personal discretion to decide whether an individual should proceed to a diagnostic test and hospitalisation or return to the community [33], these documented BEBOV clinical manifestations could potentially assist future FHF clinical case identification efforts. For example, the supposed filovirus disease hallmarks (fever plus haemorrhage) were observed relatively infrequently (3/26; 12%) in this study population. Three individuals prior to hospitalization reported fever plus haemorrhage, no individual whose axillary temperature was recorded at presentation to the Ebola ward had fever, and one or more haemorrhagic symptom was observed in only five hospitalised patients. The most frequent clinically observed symptoms in this study population (i.e. severe headache, asthenia, myalgia) are subjective and could equally indicate typhoid, shigellosis, or other endemic diseases. In outbreak settings, authors recommend continued rigorous study of human FHF clinical manifestations to increase the accuracy of clinical detection of filovirus infection, a crucial aspect of outbreak control [28]–[33]. Further improvements to diagnostic accuracy beyond that achievable through clinical and epidemiological data will require the consistent and timely dispatch of field laboratories to filovirus outbreak settings and an eventual development of a bedside diagnostic (e.g. dipstick test).

Comprehensive documentation and understanding of FHF clinical manifestations is needed, as the administration of treatment regimens should be based on presentation, anticipated symptomatology, and disease severity. Authors recommend that future clinical reporting employ an improved version of the report form, further justification of which is delineated below [19].

### Case management

Data indicate that components of standard treatment were not comprehensively administered and monitoring was infrequent or non-existent. All patients should have received antibiotics and antimalarials, but these were recorded for only 27% and 42% of patients respectively. Data preclude determining whether this was warranted or rather demonstrate sub-standard treatment, incomplete data recording, or both. Lack of recorded data on symptoms was associated with 74% increased odds of death, suggesting a possible relationship between data recording and outcome, though statistical significance was not reached. (OR 1.74; 95%CI: 0.23–15.06).

Measurement of axillary body temperature, a basic non-invasive procedure, was recorded sporadically, if at all. Only 19% (5/26) of patients had axillary body temperature recorded at least once per day for 80% of their hospital stay. This indication of sub-standard patient monitoring affects interpretation of the finding that only one patient developed fever for one day during hospitalisation. It is questionable whether this accurately reflects fever frequency for BEBOV infection, which seems likely to have been more frequent than data indicate. Sub-standard patient monitoring is also discernable from the lack of heart rate, respiratory rate, blood pressure and laboratory-based biochemical patient monitoring data. Since the use of stethoscopes and sphygmomanometers was prevented by safety concerns, it is important to develop a protocol for safe usage of such basic monitoring tools on a filovirus ward. Authors advocate for a high-biosafety field laboratory to be located near-by future outbreak epicentres so that diagnostic results are available within hours. It would also be highly desirable if laboratory testing additionally included the monitoring of patient's biochemical parameters. Patient monitoring must improve substantially if treatment regimens are to incorporate additional elements of intensive care (e.g. correcting electrolyte and metabolic imbalances, managing goal-directed haemodynamics, supplementing oxygen, and mitigating strong inflammatory responses and disseminated intravascular coagulation) or be subjected to rigorous evaluation.

While antibiotics and antimalarial administration and standard patient monitoring are deliverables for all patients, other treatment components are administered as indicated by symptomatology or disease severity. However, as in other data collection initiatives [19], we lack disease severity data. For example, did only 69% of patients experience mild pain and appropriately receive paracetamol for its alleviation, or did other patients also experience pain and not receive pain relief? This difficulty in interpretation also applies to administration of cimetidine, IV-fluids, and ORS received by 3%, 15%, and 61% of patients, respectively. Organizations responsible for filovirus patient management should, prior to the next outbreak, consider these shortcomings and improve standardised data collection accordingly.

### Study limitations

Data were collected from laboratory-confirmed EBOV hospitalised patients on the two Ebola wards and not from the additional 30 laboratory-confirmed cases identified in the community [69]. Individuals receiving hospital care may differ from those not seeking and receiving such care. For example, the latter may have less severe disease and survive more frequently, thus explaining the overall lower CFR compared to the one observed in hospitalised laboratory-confirmed patients (25% versus 42%). Moreover, limited data were collected from patients hospitalised early in the outbreak, as clinical data collection was not a priority.

Authors were limited to describing administered treatment rather than assessing its impact on clinical outcome. The small sample size and incomplete monitoring data precluded adjusting for potential confounding (e.g. by disease severity) of any association between treatment and clinical outcome. For instance, the borderline association of antimalarial treatment with fatal outcome could be explained by patients receiving this treatment being more severely ill with malaria or Ebola than those who did not.

### Recommendations for improving data collection in filovirus outbreaks

Likely reasons for incomplete patient monitoring and possible sub-standard treatment delivery include: (i) non-prioritization of systematic clinical data collection due to heavy workloads, particularly during the height of the outbreak, (ii) recording of patient data on multiple forms or blank paper, increasing likelihood of data mismanagement or loss, and (iii) lack of staff awareness of the importance and reasons for clinical data recording and supportive case management during filovirus outbreaks. Organizations responsible for filovirus patient management thus need clear and concise guidelines, training, and supplies to improve data collection and case management components, similar to those used for intensive care patients in industrialized countries.

Those responsible for filovirus case management must ensure that sufficient supplies and equipment (e.g. thermometer, timepiece measuring seconds, stethoscope, and sphygmomanometer, as used for previous filovirus patients [9], [19], [43], [47], [55], [67]) and standardised data collection forms are available at outbreak response initiation. Together with appropriate training and comprehensive supportive treatment, appropriate supplies would facilitate clinicians' efforts to deliver optimal care to future patients and enhance analyses of accurate epidemiological and clinical data, both crucial for advancing outbreak control and treatment efforts for poorly understood filovirus diseases.

Finally, laboratory tests (e.g. haemoglobin, complete and white blood cell counts) performed in past outbreaks [9], [19], [43], [47], [55], [67], should be included in routine filovirus patient monitoring. On-site laboratory capacity would greatly facilitate case management efforts through the provision of timely diagnostic and patient status results. The authors recommend on-site diagnostic and biochemical laboratory capacity where possible in subsequent outbreaks [29], [33], [34].

## Conclusions

Authors did not find important differences between the symptomatology of BEBOV and other FHF strains. Results did not confirm the remarkably low case fatality reported initially [8], but are similar to MacNeil and colleague's findings among confirmed BEBOV cases [69]. Experiencing any haemorrhagic symptom significantly increased the probability of patient death. Each additional symptom increased the odds of death, suggesting that total symptom load is a risk factor.

Recordkeeping and data collection were poor in both makeshift and fully functional Ebola wards. Standardising and strengthening data collection and recordkeeping on Ebola wards will help address the uncertainties discussed in this paper. Improved documentation and monitoring is a prerequisite for intensifying supportive care in future outbreaks. Safety protocols should be reviewed where they appear to compromise patient monitoring and care without significantly improving safety.
